# High prevalence of drug-resistance mutations in *Plasmodium falciparum *and *Plasmodium vivax *in southern Ethiopia

**DOI:** 10.1186/1475-2875-5-54

**Published:** 2006-07-03

**Authors:** Mirjam Schunk, Wondimagegn P Kumma, Isabel Barreto Miranda, Maha E Osman, Susanne Roewer, Abraham Alano, Thomas Löscher, Ulrich Bienzle, Frank P Mockenhaupt

**Affiliations:** 1 Department of Infectious Diseases and Tropical Medicine, University of Munich, Leopoldstraße 5, 80802 Munich, Germany; 2 Dilla College of Teachers Education and Health Sciences, Debub University, PO Box 419, Awassa, Ethiopia; 3 Malaria Research Centre (MalRC), Department of Biochemistry, Faculty of Medicine, University of Khartoum, P.O. Box 102, Khartoum, Sudan; 4 Institute of Tropical Medicine and International Health, Charité – University Medicine Berlin, Spandauer Damm 130, 14050 Berlin, Germany

## Abstract

**Background:**

In Ethiopia, malaria is caused by both *Plasmodium falciparum *and *Plasmodium vivax*. Drug resistance of *P. falciparum *to sulfadoxine-pyrimethamine (SP) and chloroquine (CQ) is frequent and intense in some areas.

**Methods:**

In 100 patients with uncomplicated malaria from Dilla, southern Ethiopia, *P. falciparum dhfr *and *dhps *mutations as well as *P. vivax dhfr *polymorphisms associated with resistance to SP and *P. falciparum pfcrt *and *pfmdr1 *mutations conferring CQ resistance were assessed.

**Results:**

*P. falciparum *and *P. vivax *were observed in 69% and 31% of the patients, respectively. *Pfdhfr *triple mutations and *pfdhfr/pfdhps *quintuple mutations occurred in 87% and 86% of *P. falciparum *isolates, respectively. *Pfcrt *T76 was seen in all and *pfmdr1 *Y86 in 81% of *P. falciparum*. The *P. vivax dhfr *core mutations N117 and R58 were present in 94% and 74%, respectively.

**Conclusion:**

These data point to an extraordinarily high frequency of drug-resistance mutations in both *P. falciparum *and *P. vivax *in southern Ethiopia, and strongly support that both SP and CQ are inadequate drugs for this region.

## Background

In Ethiopia, malaria is endemic in three quarters of the national territory [1] with *Plasmodium falciparum *predominating over *Plasmodium vivax*. Some 13–28% of deaths in children under five years of age are attributed to falciparum malaria [2]. Recently, a considerable increase in malaria morbidity has been noted in the south and south-west of Ethiopia. Possible reasons include climatic changes, drug resistance and migration (e.g. from Sudan). Also, epidemic outbreaks are now observed in highland areas [3].

In 1998, intense resistance of *P. falciparum *to chloroquine (CQ) necessitated a change to sulfadoxine-pyrimethamine (SP) as first-line antimalarial drug in Ethiopia. However, recent data show a high mean SP treatment failure rate of 72% in some areas [4]. Consequently, another change to artemether-lumefantrine (AL) was suggested in 2004 [5]. Yet, availability of AL is limited and 85% of the population are living in rural areas with restricted access to health care giving rise to a high rate of presumptive treatment with available drugs like CQ or SP [6].

CQ resistance is associated with a T76 mutation of the *P. falciparum *chloroquine resistance transporter gene (*pfcrt*) [7] while a multidrug resistance analogue (*pfmdr1*) Y86 variation may modulate its degree [8]. *P. falciparum *resistance to SP is conferred by mutations of the dihydropteroate synthase (*pfdhps*) and dihydrofolate reductase (*pfdhfr*) genes [9]. In East Africa, the *pfdhfr/pfdhps *quintuple mutation (triple *pfdhfr *(N108-I51-R59) *+ pfdhps *G437 *+ *E540) is predictive for SP treatment failure, whereas in West Africa the *pfdhps *alleles currently do not seem to substantially improve the predictive value of the *pfdhfr *triple mutation [10,11].

*P. vivax *has long been considered intrinsically resistant to pyrimethamine [12]. Nevertheless, *pvdhfr *mutations N117 and R58 corresponding to *P. falciparum dhfr *mutations 108 and 59, respectively, are linked with SP resistance and seem to arise first under drug pressure [13,14]. A T117 mutation is seen in highly mutated genes [14]. Both double (N117-R58) and triple *pvdhfr *mutations (N117-R58-L57) are associated with delayed parasite clearance following SP treatment [13].

Here, frequencies of relevant mutations in *pfcrt*, *pfmdr1*, *pfdhfr*, *pfdhps*, and *pvdhfr *among isolates from southern Ethiopia are presented.

## Methods

The study was conducted in Dilla (population, approximately 15,000) located in a region characterized by the gentle slopes of the southern Ethiopian rift valley system ranging between an altitude of 1,400 to 2,000 metres above sea level. The mean annual temperature ranges between 17°C and 22.4°C, and the mean annual rainfall is between 1200 and 1800 mm occurring in two rainy seasons from March to May and from July to December [15]. Both *P. falciparum *and *P. vivax *are endemic with pronounced seasonal peaks. Subjects were enrolled from January to March 2004, i.e. during high transmission at the end of a delayed rainy season. The study protocol was reviewed and approved by the Regional Government Health Bureau, Southern Nations, Ethiopia, and by the Ethical Committee, Charité-University Medicine, Berlin, Germany, and informed consent was obtained from patients or parents.

One hundred patients presenting for primary health care with fever or a history of fever and microscopically confirmed uncomplicated malaria were recruited. Treatment followed national guidelines [16]. Finger-prick blood samples were collected and malaria parasites were counted on Giemsa-stained thick blood films per 200 white blood cells (WBC). Parasite density was calculated based on a putative mean of 8000 WBC/μL. Aliquots of 10 μL of capillary blood were spotted on Whatman 3 MM filter paper, and DNA was extracted by commercial kits (QIAmp blood kit, Qiagen). Parasite species was confirmed by nested polymerase chain reaction (PCR) assays [17]. Restriction fragment length polymorphisms of PCR-generated amplicons were applied to characterize the codons: *P. falciparum dhfr *51, 59, 108, *pfdhps *436, 437, 540, 581, 613, *pfcrt *76, *pfmdr1 *86, and *P. vivax dhfr *57, 58, 61, 117. Restriction enzymes and conditions are described elsewhere [18-20,13,14]. Laboratory strains and DNA-samples of known genotype were used as positive controls. Mixed alleles (both wildtype and mutant alleles present) were considered mutant.

## Results

The median age of the 100 patients (57 female, 43 male) was 17 years (range 8 months to 60 years). *P. falciparum *mono-infection, *P. vivax *mono-infection, and *P. falciparum-P. vivax *co-infection were seen in 66, 28, and 3 patients, respectively. In three samples, PCR yielded no result and at re-examination of thick films, no parasites were observed. The geometric mean parasite density of *P. falciparum *and *P. vivax *positive specimens was 21,797/μL (95% confidence interval, 7,600–48,000) and 5,280/μL (95% confidence interval, 880–16,800), respectively.

The results of *P. falciparum *genotyping for drug resistance mutations are summarized in Table 1. All isolates showed the CQ resistance *pfcrt *T76 core mutation and four out of five additionally *pfmdr1 *Y86. Likewise, all isolates exhibited the *pfdhfr *core mutation N108 and no wildtype alleles at all three loci examined. In particular, the *pfdhfr *triple mutation was seen in almost 90% (Table 1). Both *pfdhps *G437 and E540 occurred in 97% whereas codons 436, 581 and 613 were all wildtype. Only two isolates displayed wildtype alleles in all *pfdhps *codons. Quadruple mutations were not detected but quintuple variants were found in 86% of *P. falciparum*-infected patients. As for *P. vivax pvdhfr*, a N117 mutation was present in 94% of the isolates, 74% of these additionally showed R58. Mutations at codons 57 and 61 were found in only one specimen each (Table 2).

**Table 1:** Prevalence of mutations conferring resistance to chloroquine and sulfadoxine-pyrimthamine in *Plasmodium falciparum *isolates from southern Ethiopia (n = 69)

Gene locus	Mutation (%)	Mixed type (%)^a^
*Pfcrt *76	69 (100%)	0 (%)
*Pfmdr1 *86	45 (65%)	11 (16%)
*Dhfr *51	67 (97%)	0 (%)
*Dhfr *108	69 (100%)	0 (%)
*Dhfr *59	62 (90%)	0 (%)
*Dhps *436	0 (0%)	0 (%)
*Dhps *437	67 (97%)	0 (%)
*Dhps *540	67 (97%)	0 (%)
*Dhps *581	0 (0%)	0 (%)
*Dhps *613	0 (0%)	0 (%)
		
***Grouped dhfr/dhps alleles***		
*Dhfr *I51+N108	7 (10%)	0 (%)
*Dhfr *R59+N108	2 (3%)	0 (%)
*Dhfr triple *(I51+R59+N108)	60 (87%)	0 (%)
*Dhfr/dhps *quadruple (*dhfr *triple + *dhps *G437)	0 (0%)	0 (%)
*Dhfr/dhps *quintuple (quadruple + *dhps *E540)	59 (86%)	0 (%)

^a ^both wildtype and mutant allele were found

**Table 2:** Prevalence of *Plasmodium vivax dihydrofolate reductase *mutations associated with resistance to sulfadoxine-pyrimthamine in isolates from southern Ethiopia (*n *= 31)

*Pvdhfr *codon	Wildtype (%)	Mutation (%)	Mixed (%)^a^
57	30 (97%)	1 (3%)	0
58	8 (26%)	21 (68%)	2 (6%)
61	30 (97%)	0	1 (3%)
117	2 (6%)	29 (94%)	0

^a ^both wildtype and mutant allele were found

## Discussion

Ethiopia comprises regions of largely differing malaria endemicity and transmission. Considering the substantial burden of disease in this country, there is a remarkable shortage on country-wide data on the efficacy of antimalarial drugs. A failure rate of 88% within two weeks following CQ treatment was reported from central Ethiopia already in 1999 [21]. A recent multi-site survey demonstrated mean SP treatment failure rates of 36% and 72% within two and four weeks of follow-up, respectively [22]. AL, in contrast, was reported to achieve adequate clinical and parasitological response in 99% [22].

In line with reports on high clinical failure rates of CQ and SP, the obtained data shows that in an area of seasonal transmission in southern Ethiopia, *P. falciparum *mutations conferring resistance to these drugs are abundant. In particular, *pfcrt *T76 and the *pfdhfr *triple mutation occurred in 100% and almost 90%, respectively. Also, *pfdhfr *G437 and E540 considered to confer high grade SP resistance in the presence of the *pfdhfr *triple mutant [10] were seen in all but two *P. falciparum *isolates. In addition, with the exception of *pfmdr1*, mixed alleles comprising both wildtype and mutation, were absent. Although only approximative evidence, this suggests a rather low multiplicity of infection according with the seasonal nature of malaria transmission in the study area.

The prevalence of *pfdhfr *triple and *pfdhfr/pfdhps *quintuple (86%) mutations found in this study is extraordinarily high. In Malawi, roughly three out of four patients infected with parasites exhibiting quintuple mutations have been reported to suffer SP treatment failure [10]. So far, the predictive value of these markers for SP treatment failure has neither been established in Ethiopia nor in the study area. Though this predictive value may show some variation between different locations [10,11], however, overall and considering respective studies in East Africa [23,10] these findings match with the extremely poor SP efficacy rates seen in this country [4]. In Jima, southern Ethiopia and some 200 km West of Dilla, 54% of *P. falciparum *isolates have recently been reported to carry the *pfdhfr/pfdhps *quintuple mutation [24] highlighting the uneven distribution of drug resistance in the country. Furthermore, the prevalence of quintuple mutants in the present study exceeds respective numbers in other African countries where SP had been used for a longer period of time [23,25]. The reason for the abundance of mutations conferring SP resistance in the study area of Dilla remains obscure but the findings definitely supports usage of an alternative first-line drug, e.g. AL.

*Pfcrt *T76, and to a lesser extent *pfmdr1 *Y86, are useful markers of CQ resistance in areas where it is low to moderate [20,26]. In the present study, the high prevalence of *pfcrt *and *pfmdr1 *mutations match the known poor efficacy of CQ in Ethiopia [21]. However, considering the retraction of the drug at least from official first-line treatment, perpetuation of resistant strains in the parasite population due to ongoing CQ usage also seems possible. Unfortunately, pre-treatment drug levels could not be assessed in the present study. Nevertheless, the obtained results on CQ resistance markers may have importance for subsequent monitoring: In areas where CQ was abandoned as first-line antimalarial, a recovery of CQ sensitivity and a decrease of *pfcrt *mutations have been demonstrated [27,28].

For *P. vivax*, high frequencies of *pvdhfr *N117 (94%) and N117-R58 (74%) variations but no T117, triple or quadruple mutations were observed. Although these multiple mutations seem to be necessary for in vivo resistance [13,14], this finding suggests widespread SP use since the former arise first under drug pressure [13,14]. Higher frequencies of *pvdhr *mutations are only found in Thailand where double and triple mutations were detected in 99% of isolates [13]. *P. vivax *substantially contributes to morbidity in Ethiopia and differentiating *P. falciparum *from *P. vivax *frequently is hampered by limited or absent microscopic facilities. As this implies SP treatment of *P. vivax *malaria, monitoring the resistance patterns seems worthwhile.

## Conclusion

In conclusion, *P. falciparum *mutations conferring resistance to CQ and SP are abundant in southern Ethiopia as are mutations in *P. vivax *associated with SP resistance. These findings strongly support the withdrawal of CQ and SP and the introduction of AL as first-line drug for the treatment of falciparum malaria. Repeated surveillance of the parasites' molecular markers will enable to monitor the development of resistance to CQ and SP in the region.

## Authors' contributions

FPM, WPW, TL and UB designed the study. WPW, TL, and UB were responsible for patient recruitment and parasitological examinations. MS, IBM, MEO, and SR did the PCR assays. MS and FPM wrote the paper with major contributions of the other authors.

## Conflict of interest

The author(s) declare that they have no competing interests.

## Financial support

Charité grant 89 539 150 (2005).
